# Design, rationale, and baseline characteristics of a cluster randomized controlled trial of pay for performance for hypertension treatment: study protocol

**DOI:** 10.1186/1748-5908-6-114

**Published:** 2011-10-03

**Authors:** Laura A Petersen, Tracy Urech, Kate Simpson, Kenneth Pietz, Sylvia J Hysong, Jochen Profit, Douglas Conrad, R Adams Dudley, Meghan Z Lutschg, Robert Petzel, LeChauncy D Woodard

**Affiliations:** 1 Health Policy and Quality Program, Michael E. DeBakey VA Medical Center Health Services Research and Development Center of Excellence, and Section for Health Services Research, Department of Medicine, Baylor College of Medicine, Houston, TX, USA; 2 University of Washington, Magnuson Health Sciences Center, Seattle, WA, USA; 3 Department of Health Services, Institute for Health Policy Studies, University of California, San Francisco, San Francisco, CA, USA; 4 Under Secretary for Health, Department of Veterans Affairs, Washington, D.C., USA

## Abstract

**Background:**

Despite compelling evidence of the benefits of treatment and well-accepted guidelines for treatment, hypertension is controlled in less than one-half of United States citizens.

**Methods/design:**

This randomized controlled trial tests whether explicit financial incentives promote the translation of guideline-recommended care for hypertension into clinical practice and improve blood pressure (BP) control in the primary care setting. Using constrained randomization, we assigned 12 Veterans Affairs hospital outpatient clinics to four study arms: physician-level incentive; group-level incentive; combination of physician and group incentives; and no incentives (control). All participants at the hospital (cluster) were assigned to the same study arm. We enrolled 83 full-time primary care physicians and 42 non-physician personnel. The intervention consisted of an educational session about guideline-recommended care for hypertension, five audit and feedback reports, and five disbursements of incentive payments. Incentive payments rewarded participants for chart-documented use of guideline-recommended antihypertensive medications, BP control, and appropriate responses to uncontrolled BP during a prior four-month performance period over the 20-month intervention. To identify potential unintended consequences of the incentives, the study team interviewed study participants, as well as non-participant primary care personnel and leadership at study sites. Chart reviews included data collection on quality measures not related to hypertension. To evaluate the persistence of the effect of the incentives, the study design includes a washout period.

**Discussion:**

We briefly describe the rationale for the interventions being studied, as well as the major design choices. Rigorous research designs such as the one described here are necessary to determine whether performance-based payment arrangements such as financial incentives result in meaningful quality improvements.

**Trial Registration:**

http://www.clinicaltrials.govNCT00302718

## Background

Despite compelling evidence of the benefits of treatment, hypertension is controlled in less than one-half of United States (US) citizens with the disease [1]. Inadequate blood pressure (BP) control results in excess morbidity and mortality from cardiac, renal, and peripheral vascular disease [2]. While some cases of poor BP control relate to patients' compliance with treatment, there is significant under-treatment of hypertension on the part of physicians. In one study, hypertension patients received less than 65% of care that was indicated for this condition [3].

Under-treatment of hypertension is puzzling, because good evidence exists about the efficacy of antihypertensive medications. Despite widespread dissemination of guidelines for treatment of hypertension (including the Seventh Report of the Joint National Committee (JNC 7) on Prevention, Detection, Evaluation, and Treatment of High Blood Pressure), and widespread comparative effectiveness trials such as the Antihypertensive and Lipid-Lowering Treatment to Prevent Heart Attack Trial (ALLHAT) [4], translation into clinical practice has been incomplete [5].

Awareness of barriers to such clinical research translation has raised enthusiasm about using novel methods, such as financial incentives, to overcome them [6]. A number of pay-for-performance programs have been implemented in the United Kingdom (UK) and the US [7,8]. However, research evidence of effectiveness of pay-for-performance programs, particularly randomized trials, is limited [9,10]. Using a cluster randomized controlled trial (RCT), we are testing the effect of explicit financial incentives to promote the translation of guideline-recommend care for hypertension into clinical practice and thereby improve BP control in the primary care setting. This trial addresses the needs of policy makers, payers, physicians, administrators, and others for information about a clinically relevant intervention in routine practice. We are not aware of other ongoing randomized trials of pay for performance directed at physicians and provider groups [11].

### Design of trial

Because our goal was to evaluate the impact of financial incentives on individual physicians as well as primary care provider groups, we implemented a cluster randomized controlled design and clustered at the facility level [12].

### Design of incentives

Should individuals, groups, or some combination receive financial incentives for their performance? One could anticipate that with group- or practice-team-level incentives, individual physicians would not capture the full returns on their individual effort to improve the quality of their care. Traditional economic theory suggests that the potential for some physicians to 'free-ride' on the efforts of others may lead many to reduce their individual efforts [10,13]. Conversely, the problem with rewarding individuals, but not the individual's team or group, is that cooperation among the group is not necessarily encouraged. Moreover, group incentives may be important in supporting and rewarding infrastructure improvements to the healthcare delivery system. Studies evaluating the chronic care model suggest that multi-disciplinary teams produce better patient outcomes [14,15]. Yet, traditional fee-for-service payment models create disincentives for making organizational changes in care delivery [15,16]. Thus, theory suggests the potential for group-level incentives to support organizational and team-based efforts to improve quality, but it is not yet known how these effects compare to individual-level incentives.

The design of incentives for quality also raises the question, what should we reward? Would incentives targeting processes (*i.e*., what clinicians do) or outcomes (*i.e*., what ultimately happens to patients) be expected to produce the highest quality? Of course, the best process-of-care measures are those for which there is evidence that better performance leads to better outcomes. But it is important to note that process-of-care measures may be more sensitive to quality differences than are measures of outcomes, because a poor outcome does not necessarily occur every time there is a quality problem. Furthermore, outcomes are often dependent upon events that are outside of the control of the clinician. For example, one can prescribe guideline-recommended medications, but if the patient is not able to adhere to these medications, the BP may not be controlled. One theoretical problem with using incentives solely based upon process-of-care measures is that physicians may attempt 'gaming' and focus solely on the measure (*i.e*., use of a guideline-recommended medication), while ignoring the intended outcome, which is the attainment of a target BP.

To avoid the problems associated with the exclusive use of process-of-care or outcome measures described above and to dampen the gaming incentive, both a process-of-care measure (use of guideline-recommended medications in patients who have no other compelling indications) and an intermediate outcome measure (achievement of the JNC 7 BP goal) will be used. This approach may avoid the pitfalls of process-of-care measures alone that can encourage gaming, while avoiding the disadvantage of basing incentives solely on outcomes that may be relatively rare or difficult to achieve and somewhat beyond the control of the provider. Thus, a combined approach potentially capitalizes upon the advantages and complementary nature of both types of quality-of-care measures [9]. The design of our trial reflects the following logic: other things being equal, process-based incentives will create stronger incentives for improvement in process (which the physician can directly control); similarly, process-based incentives may also produce better outcomes (assuming that the processes being incentivized improve outcomes). We believe that combining outcome-based with process-based incentives has the potential to produce even greater quality improvement than process measures alone [17].

A third design question is whether the incentive payments should be distributed according to relative performance (*i.e*., the participant's percentile ranking compared to peers) or absolute performance (*i.e*., strictly according to performance relative to the same standard)? The former approach may be problematic because participants cannot directly control the behavior or outcomes of others on whom a relative comparison is based. The latter approach rewards behavior that the individual or group can directly control [10,13]. Therefore, we designed our incentives to reward participants each time they provided guideline-recommended medications and/or their patient met the guideline-recommended BP threshold or, in the absence of controlled BP, the participant appropriately responded to uncontrolled BP [17].

In this article, we describe the methods used to assess the effects of physician- and group-level financial incentives on processes and outcomes of care for outpatients with hypertension.

## Methods/design

### Study objectives

The goals of this study were to: determine the effect of physician-level financial incentives on processes and outcomes of care for outpatients with hypertension; assess the impact of group-level financial incentives; ascertain whether there are additive effects of physician- plus group-level financial incentives; evaluate the persistence of the effect of financial incentives; and identify any unintended consequences of these explicit financial incentives.

### Study design

This study is a four-arm cluster RCT of individual physician incentives, group-level financial incentives, and performance audit and feedback to improve the translation of guideline-recommended care for hypertension in the primary care setting. Participants at study sites randomized to one of the three intervention arms earned either physician-level, group-level, or physician- and group-level incentives (combined incentive arm). Both intervention and control arm (no incentives) participants received audit and feedback reports.

### Study sites and constrained randomization

We partnered with five Veterans Affairs (VA) regional networks to implement the study. Within these networks, hospital-based VA outpatient clinics that could conduct human subjects' research and had eight or more eligible primary care physicians were potential study sites. Of the 22 potential study sites, only 12 met the requirements for study implementation: hospital director approval; Institutional Review Board (IRB) and VA Research and Development (R&D) approval; having a credentialed individual willing to serve as the site's PI; and having at least five consented physician participants at time of randomization. We stratified these 12 study sites on the following characteristics expected *a priori *to be associated with responses to financial incentives and the study outcomes of interest: teaching status, geographic location, participation in the ALLHAT study [4] (a very large trial of various medications used to treat hypertension in both VA and non-VA settings that included intensive education regarding hypertension control and use of evidence-based hypertension treatment at participating sites), and the degree of clinic geographic proximity within the primary care setting at each study site. We identified a hospital as a teaching facility if it was listed in the Association of American Medical College's (AAMC) Council of Teaching Hospitals (COTH) directory or if the American Medical Association's (AMA) Fellowship and Residency Electronic Interactive Database (FREIDA) listed the VA facility as having a 'major' affiliation with a medical school. To determine geographic region, we used US Census Bureau information to identify the corresponding Census Division for each site. Study site investigators provided clinic layout information, and we designated sites as being integrated if the layout was amenable to group cohesion (*e.g*., the primary care clinic offices were located on the same floor at the study site).

**Figure 1:**
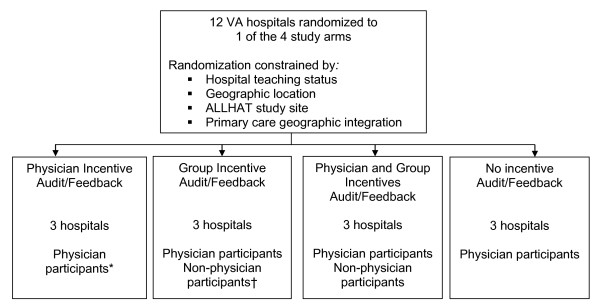
**Randomization schematic**. ALLHAT = Antihypertensive and Lipid-Lowering Treatment to Prevent Heart Attack Trial; VA = Veterans Administration. *Enrolled up to seven primary care physicians at each study site. †Enrolled up to 15 non-physician participants (*e.g*., nurses and pharmacists) at each study site.

We randomized at the cluster (facility) level. To ensure that facilities of the same type would not be concentrated in the same arm, we employed the following constraints: all non-teaching facilities could not be in the same arm; all non-ALLHAT sites could not be in the same study arm; no arm could have two sites from the same geographic location; and at least two sites per arm had to meet the criteria for geographic integration (Figure 1). Using SAS version 9.1.3 (SAS Institute Inc, Cary, NC), a data analyst on the study team who was not involved in the processes of site selection or subject recruitment assigned a uniform random number to each of the possible allocations and selected the allocation sequence with the highest random number. Table 1 lists the characteristics of the study sites.

**Table 1:** Study site characteristics

VA hospital	City, State	Teaching facility*	US Census Division	ALLHAT study site	Primary care geographic integration†
VA Boston HCS	Boston, MA	X	New England		X

Providence VAMC	Providence, RI	X	New England	X	X

VA Connecticut HCS	Newington, CT		New England		X

Charlie Norwood VAMC	Augusta, GA	X	South Atlantic	X	X

Ralph H. Johnson VAMC	Charleston, SC	X	South Atlantic	X	X

Birmingham VAMC	Birmingham, AL	X	E. South Central		X

Aleda E. Lutz VAMC	Saginaw, MI		E. North Central		

John D. Dingell VAMC	Detroit, MI	X	E. North Central	X	X

G.V. (Sonny) Montgomery VAMC	Jackson, MS	X	E. South Central	X	

Michael E. DeBakey VAMC	Houston, TX	X	W. South Central	X	

Oklahoma City VAMC	Oklahoma City, OK	X	W. South Central	X	X

Minneapolis VAMC	Minneapolis, MN	X	W. North Central	X	

ALLHAT = Antihypertensive and Lipid-Lowering Treatment to Prevent Heart Attack Trial; HCS = healthcare system; US = United States;

VA = Veterans Administration; VAMC = Veterans Affairs Medical Center

*Designated as a teaching facility if the facility was either listed in the Association of American Medical College's (AAMC) Council of Teaching Hospitals (COTH) directory or if the American Medical Association's (AMA) Fellowship and Residency Electronic Interactive Database (FREIDA) database listed the VA facility as having a 'major' affiliation with a medical school.

†Designated as geographically integrated if the primary care clinic layout was amenable to group cohesion (*e.g*., the primary care clinic offices located on the same floor at the study site).

### Power and sample size

The anticipated effect sizes were based upon a systematic review of financial incentives in healthcare [9]. In that review, the range of increases in performance using physician-level incentives was 5.9% to 25.3%, most of which were for preventive services such as immunizations. Because this study assesses treatment of a chronic disease requiring multiple types of interventions (*i.e*., medications, counseling, monitoring, lifestyle modification), rather than a single immunization, the increase in performance is anticipated to be somewhat less than those trials at the high end of this range.

The sample size calculation must take into account several factors. First, this is a cluster design, randomized at the facility level. Second, there is a measurement error associated with estimating each physician's proportion, because we can only sample a finite number of patients per physician. Finally, there must be a correction for multiple comparisons because we are testing two effects and an interaction. The sample size computation uses the non-central t-distribution and is based on a SAS^® ^program from Donner and Klar [18]. An iterative solution is required because the number of degrees of freedom depends on the sample size. The variance in measurements is the sum of the variance between hospitals, the remaining variation between physicians and the error variance of the measurement. Estimates of the variance between hospitals and the variance between physicians were obtained from pilot data. The error variance of measurement of the physician's proportion was calculated from the binomial distribution. Using these data, we estimated values of the intraclass correlation of 0.39 for appropriate medication and 0.14 for BP control.

We provide power calculations for the process and outcome measures. We calculated effect sizes for various values of the difference in percentage use of appropriate medication and BP control we could detect between the study arms with 80% power using a two-sided t-test with 95% significance. Greater increases in power result from increasing the number of clusters than by increasing the number of cases within clusters [18]. However, it is much more difficult and costly to recruit more hospitals. We chose to use three hospitals per study arm with five physicians per hospital and 40 patient charts per physician. We determined that we could detect a difference of 17 percentage points between the mean proportions of appropriate medications in the arms, for an effect size of 1.59. Similarly, for BP control, we determined we could detect a difference of 15 percentage points between the mean proportions in the study arms for an effect size of 1.30.

We adjusted the sample size to account for anticipated physician participant attrition. Using VA physician workforce planning data, we estimated 5.8% would leave VA employment during the study. We adjusted our initial sample size, five physicians per site, to seven per site to account for attrition using the formula proposed by Lachin [19], defined as 1/(1-R)^2 ^where R is the drop-out rate.

### Baseline characteristics of study participants

Primary care physicians who worked at least 0.6 full-time equivalents (approximately three days per week related to clinical activities) or had a panel size of at least 500 patients were eligible to participate. Research assistants on the Houston coordinating center study team obtained informed consent from at least five eligible participants (see Power and sample size section) at each study site prior to randomization. We sought to avoid coercion by informing the individuals that participation was strictly voluntary, that their decision to participate would not impact their employment status, and that their supervisors and other hospital officials would not have access to their performance data. Following randomization of study sites to study arms, we continued to consent eligible physician participants as necessary to meet our recruitment goal of seven physicians per site (see Power and sample size section). We enrolled a total of 83 physicians from 12 VA hospitals. At two sites, more than seven eligible physicians consented for the study, so we randomly chose seven among those who consented.

All enrolled physicians in a hospital were placed in the same study arm. At the six study sites randomized to the group-level incentive, the physicians invited up to 15 non-physician colleagues, either other clinicians (*e.g*., nurses and pharmacists) or administrative support staff (*e.g*., clerks) or both, to participate. Research assistants on the Houston coordinating center study team obtained informed consent from 42 non-physician participants. Table 2 lists the demographic characteristics of the physician and non-physician primary care personnel who were enrolled at the start of the intervention period.

**Table 2:** Characteristics of physicians and non-physician primary care personnel enrolled at the start of the intervention

Characteristic*	Primary care physicians (n = 83)	Non-physician personnel (n = 42)
Male, n (%)	45 (54.2)	6 (14.3)

Age at start of study, mean (SD), *y*	46.5 (7.8)	48.7 (8.7)

Race/ethnicity, n (%)		
White	35 (42.2)	24 (57.1)
Black	6 (7.2)	12 (28.6)
Asian	34 (41.0)	3 (7.1)
Hispanic	4 (4.8)	1 (2.4)
Other†	3 (3.6)	2 (4.8)
Unknown‡	1 (1.2)	0

Board certified, n (%)	76 (91.6)	N/A

Primary specialty internal medicine, n (%)	71 (85.5)	N/A

Trained in a subspecialty/secondary specialty, n (%)	18 (21.7)	N/A

Years practicing since residency completion, mean (SD)	12.6 (7.8)	N/A

Proportion of professional time spent delivering patient care, mean (SD)	0.89 (0.13)	0.86 (0.20)

Role in patient care delivery, n (%)	N/A	
Licensed practical nurse	N/A	10 (23.8)
Medical support assistant	N/A	3 (7.1)
Pharmacist	N/A	2 (4.8)
Physician assistant or nurse practitioner	N/A	5 (11.9)
Registered nurse	N/A	16 (38.1)
Registered nurse case manager or care coordinator	N/A	6 (14.3)

*Data collected as part of the participant demographic questionnaire conducted following the informed consent discussion.

†Other includes participants that described themselves as Native Hawaiian/Other Pacific Islander, American Indian/Alaska Native, or belonging to more than one race/ethnicity group.

‡Participant declined to answer.

### Interventions

#### Provider education

Between February and April 2008, we provided all participants with the current JNC 7 hypertension guidelines during live educational webinars presented by a cardiologist and an internist. At the sessions, participants learned their study arm assignment. Participants in the intervention arms received information about the magnitude and criteria for the incentives.

#### Audit and feedback

**Figure 2:**
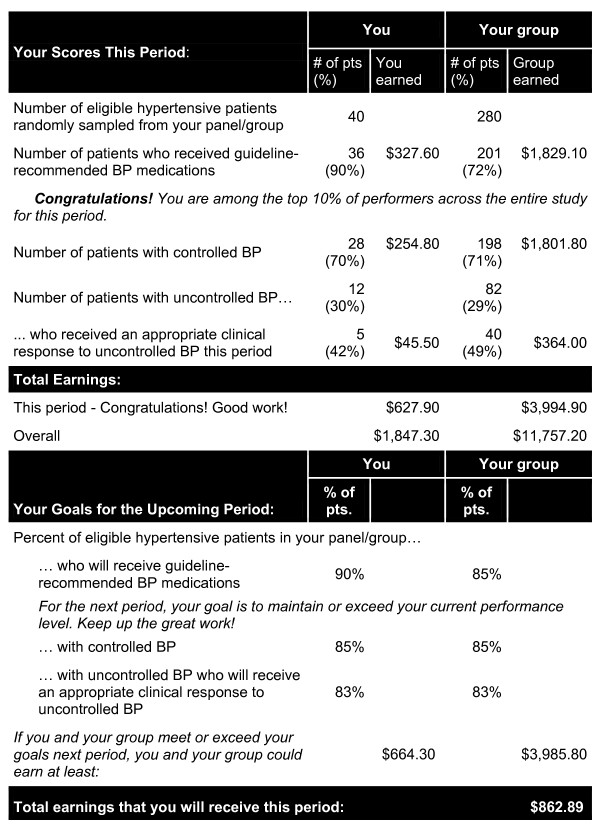
**Feedback report #3 for a physician participant in the individual and group incentives study arm**. BP = blood pressure.

All participants (including the control group) received an audit and feedback report via a secure, password-protected study website following the end of each of five data collection periods, approximately four months apart. Reports were designed to contain features, which in previous research had improved the effectiveness of feedback in healthcare settings [20,21]. Reports included data reflecting individual and group scores, earnings for the study period, total earnings to date, and future performance goals. Each time we posted the feedback report for a particular period to the website, we emailed study participants to announce its availability. Figure 2 shows the layout of the audit and feedback report for a physician participant in the combined incentive arm.

#### Financial incentives

The financial incentive intervention phase of the study started in April 2008 and consisted of disbursements of incentive payments earned over each of five performance periods. For each performance period, trained chart abstractors extracted relevant data from the VA electronic medical records of 40 randomly selected patients in each physician's panel who met eligibility criteria. Only those patients with hypertension who had a face-to-face healthcare encounter where hypertension could be addressed during the performance interval were eligible for inclusion in the sampling frame. For the first performance period, we examined a four-month period prior to the start of the incentive intervention phase to assess participants' baseline performance. We anchored performance periods two to five from the start of the incentive intervention phase. Incentive payments arrived in participants' VA paychecks approximately every four months and typically followed the posting of the feedback report for that performance period. After each data collection period, we notified participants via e-mail the date of the paycheck in which the payment was to appear.

#### Study outcomes

**Figure 3:**
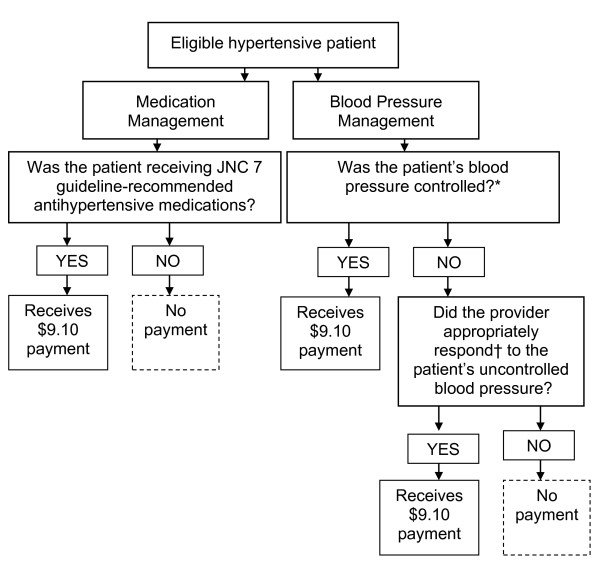
**Study outcomes assessed to determine incentive payment**. JNC 7 = Seventh Report of the Joint National Committee on Prevention, Detection, Evaluation, and Treatment of High Blood Pressure. *Blood pressure control for patients without co-existing diabetes defined as < 140/90 mm Hg; with co-existing diabetes < 130/80 mm Hg. †Defined as either adding new or increasing current antihypertensive medication, prescribing lifestyle modifications, or rechecking the patient's blood pressure within six weeks to determine if blood pressure controlled; if not controlled, responding with at least one of above actions.

We rewarded participants for delivering guideline-concordant care for management of hypertension. Incentive earnings for each four-month performance period were based on the proportion of the physician's sampled patients meeting either or both performance measures: receiving guideline-recommended antihypertensive medications; and achieving the guideline-recommended BP threshold OR having an appropriate response to uncontrolled BP (Figure 3). According to the guideline in place at the time of the study, thiazide diuretics should be used either alone or in combination with other classes of medications to control BP in most patients with uncomplicated hypertension. In patients with compelling indications for the use of other antihypertensive drug classes (*e.g*., diabetes mellitus or renal disease), medications appropriate for those high-risk conditions should be used as initial therapy for controlling BP [2]. JNC 7 guidelines recommend treating BP to a goal of < 140/90 mm Hg in most patients. In those with hypertension and co-existing diabetes mellitus and/or renal disease, the goal was < 130/80 mm Hg. Examples of appropriate responses to uncontrolled BP included increasing the dosage of a guideline-recommended antihypertensive medication or recommending a lifestyle modification, such as the Dietary Approaches to Stop Hypertension (DASH) eating plan to a patient with Stage 1 hypertension.

#### Incentive payment structure

The five participating VA Networks' contributions to the incentive fund totalled $250,000. Based on this amount, we then simulated study results using pilot data and estimated rates of improvement to determine the greatest per-outcome incentive that we could provide per physician. The results indicated a per-patient maximum amount of $18.20, one-half from use of guideline-recommended medications and one-half from either BP control or appropriate response to uncontrolled BP.

Table 3 describes the intervention components and estimated incentive payouts for the study arms. For the physician-level incentive arm, each physician's reward reflected the number of successful outcomes achieved among the 40 randomly chosen patients. The group incentive earnings were based on the aggregated performance of the physicians. We allowed participants at the six group sites to choose how to allocate the group earnings (*e.g*., divide earnings among themselves as personal income or use them to provide patient education materials or other supplies for the clinic). Each group chose to divide the earnings evenly amongst themselves. For the combined incentive arm, the physicians received a payment that included their individual performance earnings as well as their share of the group's aggregated performance reward. Non-physician participants received their share of the group's aggregated performance reward.

**Table 3:** Intervention components and incentive payouts

	Intervention components			Participants				Total estimated* incentive over entire study period	
				
Study Arm	Audit/ feedback report	Individual physician incentive	Group incentive	Physicians	Non- physicians	Performance evaluated	Per outcome per patient incentive award	Individual Physician	Group†
Physician incentive	•	•		•		Physician	$9.10	$2681	N/A

Group incentive	•		•	•	•	Physician	$9.10	N/A	$17206

Physician and group incentives	•	•	•	•	•	Physician	$9.10	$2696	$18872

No incentives (control)	•			•		Physician	$9.10	N/A	N/A

*Estimates based on outcome rates from pilot study and projected improvement rates per period as follows: individual arm - 0.05 for process measures (guideline-recommendation medication and appropriate response) and 0.03 for blood pressure control; group arm - 0.02 for process measures and 0.01 for blood pressure control; combined arm - 0.06 for process measures and 0.04 for blood pressure control.

†For a group that includes seven physicians. Group incentive award determined by summing the incentive amounts earned by all seven physicians in the group.

### Data collection

We developed a data abstraction tool and electronically collected data on the physicians' patients from the VA Computerized Patient Records System (CPRS). Abstractors at the Houston coordinating center collected data on patient demographics, vital signs, diabetes mellitus, cardiovascular conditions, renal conditions, terminal illness, and other relevant co-morbidities, laboratory values and medications, including dosages, allergies, contraindications, and patient refusals for all study sites. Additionally, we collected lifestyle modifications recommended by the provider or other medical staff members and information on study outcomes addressed at follow-up visits within a given timeframe. We collected hemoglobin A1c (HbA1c) levels in patients with diabetes, low-density lipoprotein cholesterol (LDL-C) levels in patients with hyperlipidemia, and colorectal cancer screening procedures among eligible patients to assess unintended consequences, *i.e*., neglect of these conditions or missed screening opportunities, that may have resulted from the intervention's focus on hypertension care.

After a training session, chart abstractors were required to complete 20 practice charts with 95% agreement on key data elements with all other abstractors and the trainer. During each period of data collection, the abstraction manager spot-checked charts for accuracy in abstracting key variables, addressing problem areas with abstracters as necessary. At the end of each period of data collection, we randomly selected at least three charts from each site in order to evaluate abstracter agreement. Also, we blinded chart abstractors to the study's objectives and to study arm assignments to ensure impartiality in the data collection process.

### Interviews - team planning, pay-for-performance perspectives, and unintended consequences of incentives

After participants received their second incentive payments and audit and feedback reports, the study team, including an industrial/organizational psychologist, interviewed 17 physician participants and 11 non-physician participants across the study sites to collect data about the strategies that they used to improve hypertension care, including clinic planning and team interaction shared mental models. Twelve physician participants and four non-physicians also completed a similar interview after the fourth disbursement of incentive payments and audit and feedback reports. These distinct interviews were intended to provide insights on how care strategies and planning may have evolved over time, and the drivers behind any observed changes.

Following the posting of the participants' final audit and feedback reports in April 2010, we interviewed 28 physicians and 10 non-physician participants across the study sites to ascertain their perspectives on financial incentives, the intervention's impact on team dynamics and care delivery for hypertension and non-related conditions, and organizational changes that occurred at the study site during the intervention period. Focusing on similar concept domains, we also interviewed primary care personnel, 24 physicians and 25 non-physicians across the sites, who did not participate in the intervention and primary care leadership at the 12 sites.

### Washout period and post-washout period data collection

**Figure 4:**
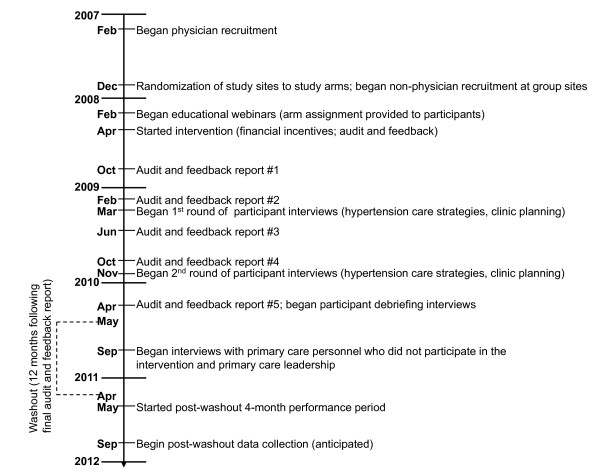
**Study timeline**.

The four-month post-washout performance period began 12 months following the posting of the final audit and feedback report to the study's website. To evaluate the persistence of the intervention, we will subsequently collect the same data that were collected during the intervention. A timeline of the study's activities, from physician recruitment to post-washout data collection, is presented in Figure 4.

### Data analysis

The unit of analysis will be the physician. We will perform a repeated measures longitudinal analysis using mixed models to evaluate the effect of the intervention. We will evaluate three different predictors for each outcome: each incentive arm versus the control arm; individual-level incentive arms versus arms with no individual incentives; and group-level incentive arms versus arms with no group incentives. Models will be developed independently for each outcome. First, using scientifically relevant covariates selected *a priori*, we will construct a maximal model for each outcome as described by Cheng *et al*. [22]. The maximal model provides the flexibility to evaluate both the covariance structure and the list of covariates for inclusion in the final model. The relationship between each continuous covariate and the outcome will be explored to assess the need for covariate transformations. We also will determine whether any site-to-site variation exists and include the facility (cluster) as a random effect as necessary. We then will perform backward elimination to delete variables of no value, arriving at our final model.

## Discussion

In this paper, we describe our rationale, methods, and baseline participant characteristics for a cluster-randomized trial to assess the effectiveness of pay for performance in improving hypertension control and use of guideline-recommended medications in the primary care setting. The potential impact of this study is great. Seventy-two million Americans have hypertension [23], but hypertension is controlled in less than one-half of those who carry this diagnosis [1]. A variety of methods, such as audit and feedback, academic detailing, reminders, guidelines, and combinations of interventions have not succeeded in eliminating the under-treatment of hypertension [24]. Given the track record of other interventions, the effectiveness and cost-effectiveness of financial incentives in overcoming such barriers to translation of research into practice must be rigorously evaluated [25].

Other studies of the use of financial incentives for improving healthcare quality have suffered from a dilution of the incentive due to multiple payers or competing incentives that make it difficult to assess effectiveness [9,26]. Relative to these situations, studying incentives in the VA healthcare system provides several advantages. First, the VA is both the insurer and the provider, so organizational responses to incentives are easier to anticipate and assess. Second, the VA uses a single payment approach, rather than a diverse mix as seen elsewhere. The VA employs salaried physicians to care for its enrollees. The VA healthcare system consists of 21 networks that operate on a global budget appropriated by Congress. Funding is distributed to the networks via a form of capitation in which payments are made per veteran meeting eligibility criteria [27]. The VA has a common national electronic medical information system, making the collection of common practice data across widely disparate geographic sites and types of facilities feasible. Thus, carrying out the study in the VA allows an evaluation of the effectiveness of financial incentives that is free from some of the limitations encountered in other settings.

Paying more for healthcare services shown to improve quality could have a tremendous impact on care delivery. However, despite great potential, numerous questions are unanswered. How effective (and cost-effective) are financial incentives for quality? Can we expect the effect of financial incentives to persist after they are stopped? Will important patient care activities that are not rewarded financially be neglected? Thus, despite enthusiasm about the potential for aligning financial incentives with high quality healthcare, there are a number of fundamental unanswered questions about their optimal design, effectiveness, and implementation that we will address in this trial [9].

While our study design has numerous strengths, we must acknowledge some limitations. First, as part of the VA Healthcare Personnel Enhancement Act of 2004 (implemented in 2006) [28], the VA healthcare system instituted a new payment system that includes performance pay based on the accomplishment of specific clinical quality goals and objectives which may be established at the local, network, or national level. However, although this program provides financial incentives for improvements in quality of care, the specific measures (*i.e*., hypertension control, diabetes management, colorectal cancer screening) for which payments are given have not been implemented uniformly across VA facilities. In addition, VA performance pay encompasses a wide array of measures, while our incentive focuses solely on management of hypertension, thus maximizing the effect of our intervention. Further, by using a RCT design, we limit the likelihood of confounding by other concurrent quality improvement programs. Second, the VA has an extensive system of clinical reminders (*i.e*., use of thiazide diuretics in patients with hypertension or use of aspirin in patients with ischemic heart disease) designed to promote the provision of guideline-recommended care. However, while the VA has implemented a variety of quality improvement strategies, our pilot data indicate that there remains room for further improvement. Third, although the VA healthcare system cares for few women patients, the subjects of this study are physicians. There is little reason to believe that the effect of a financial incentive to a physician would result in different treatment of a woman or a man with hypertension. Thus, findings from this study will be relatively generalizable to budgeted systems and staff-model health maintenance organizations (HMOs) that serve many millions of patients, to Centers for Medicare and Medicaid Services (CMS) with 40.5 million beneficiaries, and indirectly applicable to other healthcare delivery models. Finally, it is possible that there may be a 'volunteer effect' among participants. For example, physicians with a bias toward use of financial incentives may have been more likely to participate in our study. However, we expect that the randomized control design of our study will limit the effect of this potential source of bias.

In this paper, we have given a brief description of the rationale for the interventions being studied in this trial of pay for performance, as well as some of the design choices. Rigorous research designs such as this one are necessary to determine whether performance-based payment arrangements result in meaningful quality improvements. In this large cluster-RCT of pay for performance, we are seeking to provide such evidence for one of the most common chronic conditions affecting US citizens.

## Competing interests

The authors declare that they have no competing interests.

## Authors' contributions

LAP conceived of the study, obtained funding, supervised the research team, and drafted the manuscript. All authors participated in designing the study. TU, KS, KP, MZL, and LDW helped to draft the manuscript. SJH, JP, DC, and RAD provided critical revision of the manuscript for important intellectual content. All authors approved the final version of the manuscript.
